# Maternal and Fetal-Placental Effects of Etanercept Treatment During Rats’ Pregnancy

**DOI:** 10.3389/fphys.2021.787369

**Published:** 2022-02-03

**Authors:** Gabriel Gomes Araujo, Rinaldo Rodrigues dos Passos Junior, Rosaline Rocha Lunardi, Gustavo Tadeu Volpato, Thaigra Sousa Soares, Fernanda Regina Giachini, Victor Vitorino Lima

**Affiliations:** ^1^Institute of Biological Sciences and Health, Federal University of Mato Grosso, Barra do Garças, Brazil; ^2^Institute of Biological Sciences, Federal University of Goias, Goiânia, Brazil

**Keywords:** etanercept, pregnancy, medication, TNF-α, placenta, malformation

## Abstract

Etanercept is a tumor necrosis factor alpha (TNF-α) inhibitor chronically used to treat autoimmune diseases. However, the use of etanercept during pregnancy still needs to be further investigated. The aim of this study is to evaluate the etanercept treatment during pregnancy, analyzing maternal reproductive performance, fetal outcomes, and placental repercussions. Wistar rats (200–250 g) were mated and randomly distributed into two experimental groups: control and etanercept (*n* = 10 animals/group). Treatments with etanercept (0.8 mg/kg, s.c.), or saline (control group) were carried out on days 0, 6, 12, and 18 of gestation. On the morning of the 21st day of pregnancy, rats were euthanized in a CO_2_ chamber and submitted to laparotomy to remove the fetuses, placentas, ovaries, and maternal organs. There were no differences between groups in the following parameters: water and food consumption; placental efficiency; reproductive parameters, including number of corpora lutea and implants, reabsorption, and pre- and post-implantation losses. However, etanercept treatment increased liver weight, reduced fetal and placental weight, decreased the placental junction zone, reduced the percentage of normal fetuses, and increased visceral or skeletal fetal abnormalities. Therefore, etanercept resulted in damages more related to fetus and placenta. However, more studies with different doses are required to better predict possible injuries elicited using etanercept during pregnancy.

## Introduction

During pregnancy, the maternal environment undergoes many physiological adaptations to enable the presence of the concept in the body, ensuring the immunotolerance to the developing fetus (Hviid et al., 2006). To achieve this, the actions of several cytokines are necessary to help the gestational process and the balance between T helper (Th) 1 cytokine profile toward the Th2 profile is crucial to a successful pregnancy (Sykes et al., 2012). For example, a pro-inflammatory environment is required during implantation and parturition (Chaouat, 2013).

Among them, tumor necrosis factor alpha (TNF-α), a Th1 cytokine, is one of the earliest and most potent mediators of the inflammatory response (Oliveira et al., 2011). In fact, Th1 cytokines including TNF-α are required for vasculogenesis during implantation (Demir et al., 2010). However, several obstetric disorders, including recurrent miscarriage, early and severe pre-eclampsia, diabetes and recurrent implantation failure syndrome, can be originated from augmented TNF-α levels (Alijotas-Reig et al., 2017). Therefore, during pregnancy, a perfect balance between Th1 and Th2 cytokines is required for a normal pregnancy outcome.

Tumor necrosis factor alpha overproduction mediates a large spectrum of diseases, including some autoimmune conditions. On this regard, TNF-α inhibitors have been successfully used to control several autoimmune diseases including ankylosing spondylitis, rheumatoid arthritis, polyarticular juvenile idiopathic arthritis, psoriasis as well as psoriatic arthritis (Beltagy et al., 2021). Most of these diseases may affect women in the reproductive period of life, raising the question regarding the safety of the use of TNF-α inhibitors during pregnancy.

Etanercept is a soluble recombinant TNF-Fc complex, formed by extracellular fragments of TNF-α receptors attached to a human IgG Fc fraction (Mouyis et al., 2019), which blocks TNF-α and is currently recommended being used until the end of the second trimester of pregnancy, according to the European League Against Rheumatism (EULAR) recommendations (Götestam Skorpen et al., 2016). TNF-α inhibitors are considered safe, when exposition occurs during pregnancy and lactation, as observed in experimental models (Beltagy et al., 2021). Similarly, some studies conducted in humans showed no evidence of augmented risk of congenital malformation when the TNF-α inhibitors were used in the first trimester (Cooper et al., 2014; Lichtenstein et al., 2018).

However, recently, it was demonstrated that some TNF-α inhibitors may display a cumulative outcome, since spontaneous abortion, preterm labor and low birth weight rates were increased when they are used during the entire pregnancy, as was the case for infliximab (Geldhof et al., 2020). With this regard, it is known that Etanercept cross the placenta in small amounts (Berthelsen et al., 2010), suggesting that some alterations may be due *trans*-placental permeability of etanercept.

Even this negative evidence, women with autoimmune disease may benefits from anti-TNF therapies. Unfortunately, due to the limited available data, the recommendations differ on the continuity or discontinuation of anti-TNF treatment during pregnancy, and discontinuation may carry a risk of relapse of the maternal disease (Gisbert et al., 2016). Etanercept is currently classified as a category B classified drug, meaning that animal reproduction studies have failed to demonstrate a risk to the fetus, but parallelly, the availability of similar studies in pregnant women is still pending. Therefore, the clinical impact of fetal exposure to etanercept is not fully elucidated.

To answer this question, we formulated the hypothesis that the use of etanercept during the gestational process impacts maternal reproductive performance, fetal and placental changes in Wistar rats. In the present study, we investigated Wistar rats submitted to etanercept treatment during pregnancy, to assess maternal reproductive performance and possible fetal and placental alterations.

## Materials and Methods

### Animals

The animals used in the research were kept in the animal facilities of the Federal University of Mato Grosso, in 12-h light/dark cycles, under controlled temperature (23 ± 1°C) and free access to food (standard chow) and water. All procedures were performed in accordance with the guidelines provided by the Brazilian College of Animal Experiments, approved by the Animal Ethics Committee (CEUA #23108.038471/2019-14) of the Federal University of Mato Grosso.

Wistar rats in reproductive age, weighing between 200 and 250 g, were used. For mating, housed in a proportion of four females to one male Wistar rats. Vaginal swabs were collected daily, and pregnancy was confirmed by the presence of sperm concurrent with the occurrence of the female estrous cycle, and this was designated the day 0 of gestation.

### Experimental Design

Pregnant Wistar rats were randomly separated into control (*n* = 10) and etanercept [Enbrel^®^, Pfizer–(*n* = 10)] groups. The treated group received a dose of etanercept (0.8 mg/kg) subcutaneously on days 0, 6, 12, and 18 of gestation (Small et al., 2016). The control group received saline solution, following the same therapeutic protocol. Food and water consumption was evaluated daily.

On the morning of the 21st day of pregnancy, the rats were killed in a CO_2_ chamber. Then, the rats were submitted to laparotomy. The maternal heart, liver, spleen, and kidneys were removed and weighed to obtain the relative weight of each organ, using the following equation: relative organ weight = (absolute organ weight/final body weight) × 100, where the final weight corresponds to rat weighted on 21st days of pregnancy, minus litter weight. Fetuses and their respective placentas were also immediately removed and individually weighed.

### Maternal Reproductive Performance

The points of implantation, reabsorption (embryonic death) and numbers of living and dead fetuses were observed and counted. The ovaries were removed for observation and counting of corpora lutea. The pre-implantation loss percentage (embryo loss rate in the period prior to implantation) was calculated as follows: (Number of corpus luteum-Number of implantation/Number of corpus luteum) × 100. The percentage of post-implantation loss (death of embryos after implantation) was calculated as follows: (Number of implantations-Number of living fetuses/Number of implantations) × 100 (Araujo-Silva et al., 2021). The placental efficiency was calculated by the ratio between fetal weight and placental weight (Hayward et al., 2016).

### Fetal Analysis

Pups from the etanercept-treated dams whose birth weights were lower (<10th percentile) or greater (>90th percentile) than the mean birth weight of the control pups were classified as small for gestational age (SGA) or large for gestational age (LGA), respectively, and reported as percentage. The mean birth weight of the control pups was 5.05 ± 0.49 g. Therefore, pups from mothers treated with etanercept with birth weights smaller than 4.40 g (<10th percentile) were considered SGA and weighting more than 5.51 g (>90th percentile), as LGA (Dallaqua et al., 2012).

After weighing the fetuses, the analysis of external anomalies was performed. Newborns were examined externally, with detailed analysis of eyes, mouth, ear implantation, cranial conformation, fore and hind limbs, anal perforation, and tail (Macedo et al., 2021). After external examination, half of the newborns from each litter were placed in Bodian’s solution to fix the visceral structures and decalcify the bones. After fixation was completed, the serial section method proposed by Wilson (1965) was used to observe visceral anomalies.

Thus, half of the newborns of each litter were conditioned in alcohol (70%), and later were eviscerated, diaphanized with potassium hydroxide and stained with alizarin. For the analysis of skeletal anomalies, the method of Staples and Schnell (1964) was used. Ossification centers were counted and analyzed using parameters proposed by Aliverti et al. (1979) to determine the degree of fetal development.

### Placental Morphometry

Placentas were removed, dived in the central region, and immediately immersed in Methacarn (methanol 60%, chloroform 30%, and acetic acid 10%) for 3 h, at a temperature of 4°C, under constant agitation. Then, the placentas were dehydrated and were clarified in xilol. Subsequently, tissue infiltration was performed in two paraffin baths, inside a heating oven adjusted to 60*^circ^*C, followed by the battery for inclusion of placentas in paraffin, left at room temperature for 24 h for paraffin solidification. Sections (4 μm) of the placenta were serially performed from the central region, with the aid of a microtome (microtome Thermo Fisher Scientific HM355S). Then, the cuts were stretched in a 50°C floating bath and were collected from the bath on glass slides previously treated with 0.1% poly-L-lysine (Sigma), for better adherence of the cuts.

The slides containing the sections were submitted to the deparaffinization process. Subsequently, they were gradually hydrated. For morphological analysis, staining with hematoxylin-eosin was performed. The slides were then dehydrated in a 95% alcohol bath and in two absolute alcohol baths, for 10 min each, and cleared in two xylene baths, for 5 min each. Finally, the slides were assembled using PERMOUNT (Fisher-SP15-500) as a means of adhesion between the slide and cover slip.

Images were observed through a microscope (Nikon Eclipse–E200) with a 4× magnification objective. Placental images were captured using a digital camera (Option ISH-500). The entire area of the junctional zone and the labyrinth was measured in mm^2^ using the IMAGE—PRO PLUS software (Media Cybernetics, Silver Spring, MD, United States).

### Statistical Analysis

Data are presented as mean ± SD and “*n*” represents the number of animals used in the experiment. Statistical analysis was performed (GraphPad Prism 5.0, Graph Pad Software Incorporated, CA, United States). The data was submitted to the Shapiro-Wilk normality test, to ensure its normal distribution. Student’s *t*-test was used to make parametric comparisons and Mann Whitney test was used for non-parametric comparisons. For percentage analysis, Fisher’s exact test was used in. Values of *p* < 0.05 were considered statistically significant.

## Results

As expected, weight gain was observed over the weeks of gestation in both groups. The relative weight (g) of the heart, spleen, and kidneys of rats did not differ between the experimental groups. However, the liver relative weight in the etanercept group was higher than in the control group (Table 1).

**TABLE 1:** Maternal physiological parameters of control and etanercept-treated rats.

Groups			
	Control (*n* = 10)	Etanercept (*n* = 10)	*P*-value
**Weight gain in pregnancy (g)**			
1st week	10.70 ± 4.99	9.50 ± 6.42*	0.88
2nd week	20.50 ± 7.04	19.60 ± 8.04	0.93
3rd week	68.40 ± 13.82	66.50 ± 15.18	0.93
Total body weight gain–BWG (g)	99.60 ± 14.80	94.60 ± 16.89	0.83
Gravid uterus weight–GUW (g)	69.27 ± 10.29	70.88 ± 14.59	0.93
BWG minus–GUW (g)	30.33 ± 12.38	23.71 ± 5.96	0.64
Daily food consumption (g)	17.65 ± 3.02	18.60 ± 2.81	0.82
Daily water intake (mL)	37.40 ± 11.12	42.86 ± 14.19	0.77
**Relative organ weight (%)**			
Heart	0.28 ± 0.03	0.27 ± 0.03	0.98
Liver	3.20 ± 0.20	3.90 ± 0.26*	0.04
Spleen	0.15 ± 0.03	0.15 ± 0.02	0.36
Kidneys	0.25 ± 0.02	0.26 ± 0.02	0.80

*Data shown as mean ± standard deviation (SD).*

*p < 0.05 vs. Control group (Student’s t-test).

**FIGURE 1:**
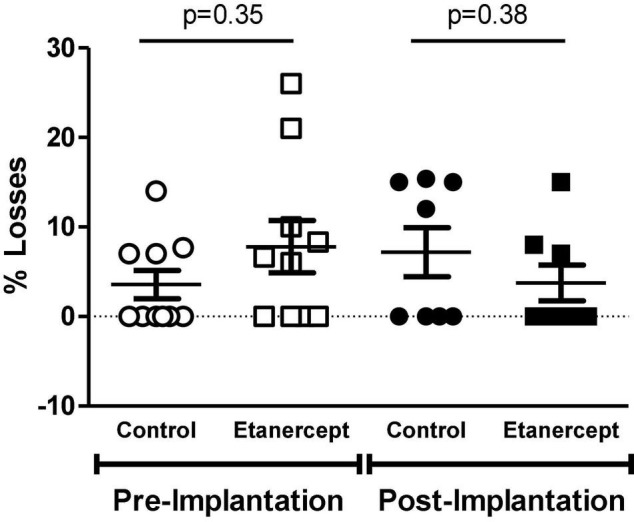
Percentage of embryo losses before and after implantation, evaluated at term of pregnancy (DP21) of control and etanercept-treated rats. Pre-implantation losses of the control group (open circles, *n* = 10) and etanercept (open squares, *n* = 10). Post-implantation losses of the control group (dark circles bar, *n* = 10) and etanercept (dark squares, *n* = 10). Statistics was conducted using the Mann Whitney test.

There was no difference between pre- and post-implantation losses between the experimental groups (Figure 1).

Table 2 brings the results related to fetal development. The fetal and placental weight of the etanercept group were decreased, compared to the control group. The number of pups born LGA was reduced in the etanercept group. In fetal development and placental efficiency there were no differences between the groups.

**TABLE 2:** Fetal and placental development and placental efficiency of control and etanercept-treated rats at term of pregnancy (day 21 of pregnancy).

Groups			
	Control (*n* = 104)	Etanercept (*n* = 111)	*P*-value
Fetal body weight (g)	5.15 ± 0.33	4.85 ± 0.22*	0.03
SGA fetuses	4.81%	7.21%	0.57
AGA fetuses	90.38%	92.79%	0.62
LGA fetuses	4.81%	0.00%*	0.03
Ossification sites	24.30 ± 2.60	23.11 ± 2.58	0.75
Placental weight (g)	0.48 ± 0.02	0.45 ± 0.03*	0.05
Placental efficiency	10.43 ± 0.33	10.73 ± 0.43	0.47

*Data shown as mean ± standard deviation (SD) and proportions (%).*

*SGA, small for gestational age; LGA, large for gestational age.*

*p < 0.05 vs. control group [Student’s t-test (mean data) or Fisher’s Exact Test (% data)].

**FIGURE 2:**
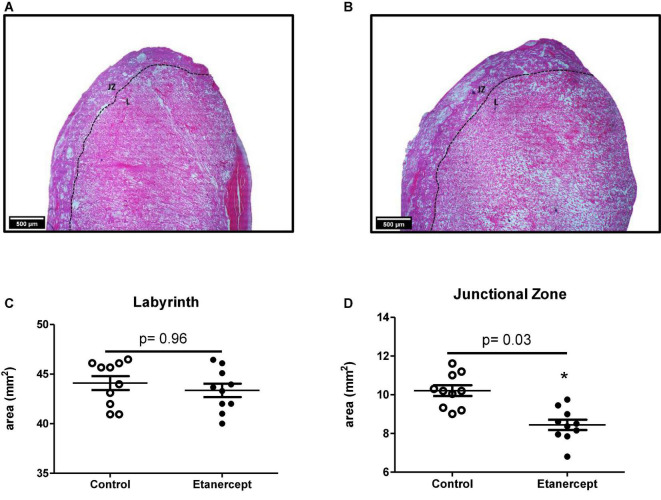
Placental area [junctional zone (ZJ) and labyrinth (L)] of control and etanercept-treated rats. Photomicrography specifying the placental regions of the control group **(A)** and etanercept-treated group **(B)**. Placental **(C)** labyrinth and junctional zone **(D)** areas (mm^2^) from the control group (open circles, *n* = 10) and etanercept-treated group (dark circles, *n* = 10). *p < 0.05 vs. control group (Student’s *t*-test).

The effects of etanercept treatment on placental area was evaluated in two distinct regions: the junctional zone and the labyrinth. The placental junctional zone of the etanercept-treated group was smaller, compared to the control group (8.37 ± 1.10 mm^2^ vs. 10.05 ± 1.28 mm^2^, respectively; *p* = 0.03). In the labyrinth, there was no difference between the control and etanercept-treated group (44.21 ± 4.24 mm^2^ vs. 44.11 ± 3.36 mm^2^, respectively; *p* = 0.96) (Figure 2).

**FIGURE 3:**
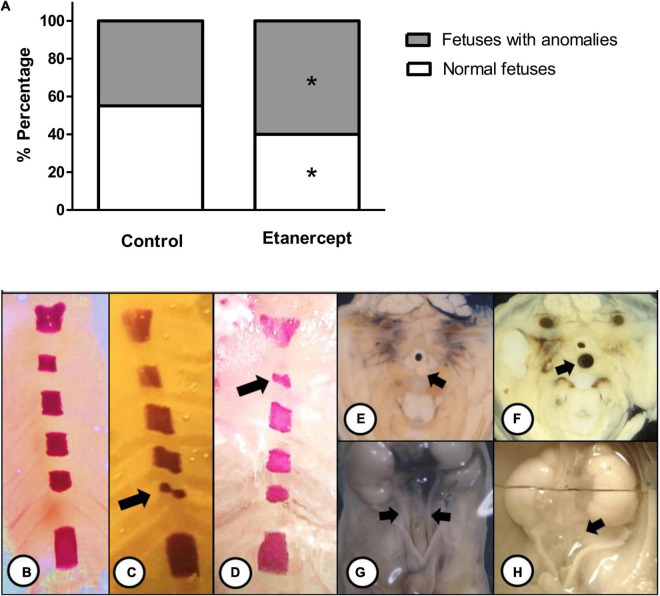
**(A)** Percentage (%) of anomalies of fetuses from control and treated etanercept during pregnancy. Panels **(B–H)** representative images of the main skeletal **(B–D)** and visceral **(E–H)** anomalies. Panel **(B)** normal sternebra of rat fetuses. Panel **(C)** abnormally shaped sternebra (arrow). Panel **(D)** incomplete ossification of sternebra (arrow). Panels **(E,F)** a thoracic section from rat fetuses, with **(E)** normal trachea (arrow) and **(F)** dilated trachea (arrow). Panels **(G,H)** the pelvis section from rat fetuses, with **(G)** normal ureter (arrow) and **(H)** enlarged ureter—hydroureter (arrow). *p < 0.05 vs. control group (Fisher’s exact test).

Figure 3 shows the most frequent skeletal and visceral anomalies including, abnormal shaped sternebra (Figure 3C), incomplete ossification of sternebra (Figure 3D), dilated trachea (Figure 3F), and enlarged ureter (Figure 3H). The percentage of normal fetuses was lower in the etanercept-treated group compared to the control group. The frequency of fetal visceral anomalies, specifying the mainly fetal occurrences is described in Table 3.

**TABLE 3:** Fetal anomalies.

Groups			
	Control	Etanercept	*P*-value
**Skeletal anomalies**			
Examined fetuses (normal/anomalies)	57/31	59/43	
Reduced rib	1 (1.7%)	2 (3.3%)	1.00
Agenesis sternebrae	1 (1.7%)	7 (11.8%)	0.06
Unossified sternebrae	3 (5.2%)	9 (15.2%)	0.13
Split sternebrae	4 (7.0%)	3 (5.0%)	0.71
Abnormally shaped sternebrae	25 (43.8%)	30 (50.8%)	0.46
**Visceral anomalies**			
Examined fetuses (normal/anomalies)	47/16	53/23	
Ureter extended	6 (12.7%)	10 (18.8%)	0.43
Calice extended	2 (4.2%)	0 (0.0%)	0.23
Distended Trachea	8 (17.0%)	17 (32.0%)	0.11

*Frequency of fetal anomalies in the control and etanercept-treated groups, specifying the anomalies found.*

*Data shown as percentage (%).*

## Discussion

The main finding of this work is that fetus from Wistar rats displayed augmented visceral and skeletal anomalies upon Etanercept-treatment. This finding raises one important concern regarding the risk/benefit profile of anti-TNF therapy during pregnancy, since this drug is widely used by women with autoimmune disease, during reproductive age.

Previous work reported that etanercept treatment improved maternal blood pressure, pregnancy outcome and uterine artery function in an experimental model of hypertension (Small et al., 2016). Besides an elegant body of data was presented in that study, it remained to be established how etanercept-treatment would impact on reproductive performance and fetal/placental outcomes upon etanercept treatment in non-hypertensive rats. Therefore, a similar dose regimen was adopted in the current study, to answer this remaining question.

Tumor necrosis factor alpha regulates the gestational process since the early stages of development, and progressively increases with the course of the pregnancy, playing an important role in the inflammatory mechanisms that regulate implantation (Alijotas-Reig et al., 2017). When evaluating maternal reproductive performance, the number of corpora lutea, implantation sites, live fetuses, and reabsorption, were similar between groups. Likewise, no difference was found in the groups between pre- and post-implantation losses, reabsorption rates and sex ratio. Therefore, treatment with etanercept, at the therapeutic dose studied, was not able to interfere with these parameters.

As pregnancy progresses, the placenta turns to the major source of TNF-α production and plays an essential role in fetal growth and support (Alijotas-Reig et al., 2017). Etanercept-treatment resulted in lighter fetuses compared to the control group, indicating that TNF-α inhibition resulted in some degree of growth restriction. Birth weight and gestational age are stronger prognostic factors for survival and life quality of a newborn (Anchieta et al., 2004). On this regard, maternal nutrition, associated with the placental capacity to transport nutrients to the fetal circulation, are major factors related to birth weight (Zhang et al., 2015) and therefore, fetal growth restriction is frequently associated with functional or structural impairment of the placenta (Sharma et al., 2016).

In the present study, etanercept treatment resulted in decreased placental weight, conversely to the higher maternal liver relative weight. Etanercept is known to cross the placental barrier, being detected in the serum of children born from patients treated with etanercept during pregnancy (Berthelsen et al., 2010). However, a recent studied reported that etanercept was not detectable in cord blood sample, whereas others TNF-α inhibitors were detectable, including adalimumab and infliximab (Ghalandari et al., 2021). Therefore, one possibility is that TNF-α inhibition may be impacting on placentation. Regarding the augmented maternal liver weight, it is well accepted that organ weight changes are accepted as a sensitive indicator of xenobiotic induced organ-damage (Lazic et al., 2020).

Placental function may be also related to morphometric evaluation, since morphological and functional placental adaptations can affect the nutrient transport capacity and consequently, placental efficiency (Sibley et al., 2010). Etanercept-treatment resulted in reduced junctional zone area, but not in the labyrinth. In rodents, the reduction in the size of the junctional zone is associated with a reduction in the number of spongiotrophoblast cells and glycogen cells (Lefebvre, 2012), which, according to Woods et al. (2018), favors systemic deleterious effects on the fetus and mother. Besides we found that etanercept resulted in reduced junctional zone, the infusion of TNF-α did not alter the junctional zone volume, as demonstrated by magnetic resonance microscopy (Bobek et al., 2018). The volume of the junctional zone expands until day 16.5 of gestation in rodents and reduces by day 18.5 (Coan et al., 2004). Some authors speculate that this effect is due migration of trophoblast glycogen cells from junctional zone to decidua and the physiological absence of TNF-α may be further impaction on this process.

Regarding fetal anomalies, the etanercept-treatment had a lower percentage of normal fetuses, which comprise individuals without any skeletal or visceral anomaly. Skeletal alterations most frequently found in newborns, such as incomplete ossification and abnormally shaped sternebra, are considered merely observed variations and have no teratogenic effect (Soares et al., 2018). Furthermore, the most frequent visceral changes found in this study were enlarged ureter (hydroureter) and dilated trachea. The increase in the ureter is an easily recognized change due to its “S” shape, which may or may not be associated with congenital hydronephrosis or any pelvic alteration and is usually transient in rodents (Taylor, 1986).

The use of TNF-α inhibitors may be useful during pregnancy, specially to conditions, were exacerbated TNF-α elicits obstetric complications. For example, the use of etanercept during pregnancy in stroke prone spontaneously hypertensive rats (SHRSP) resulted in important gestational gains such as reduced maternal blood pressure, partial reversion of endothelial dysfunction and improvement of gestational parameters including, reversing growth restriction, and restoring placental morphology (Small et al., 2016). This is probably related to the high levels of TNF-α observed during hypertension. Opposing some of these results, in our hands, etanercept resulted in a less robust fetal growth, with reduced LGA fetus, but the animals used in the current study are normotensive rats, and TNF-α levels are not pathologically augmented. Our group recently demonstrated that etanercept partially reduced blood pressure in spontaneously hypertensive rats (SHR), a model where all fetuses were classified as SGA and the mother displayed increased TNF-α levels, but etanercept was not able to restore fetal growth in SHR (Dos Passos Junior et al., 2021).

Compared to other pharmacological therapies used to reduce TNF-α actions, including monoclonal antibodies, only a small portion of etanercept cross the placenta (Romanowska-Próchnicka et al., 2021). Even in small amounts, etanercept cross the placental barrier in humans, raising the possibility that part of the alterations reported here may be due the *trans*-placental permeability of etanercept. Considering the importance of TNF-α for the gestational process, it is important to further address how the *trans*-placental transport of etanercept could impact pregnancy.

The most important limitations of the current study are that a unique dose was evaluated, the etanercept-treatment before and being continued during pregnancy was not performed; and the detection of maternal and fetal blood levels of etanercept were not addressed. It is possible that treatments being performed with doses higher than the one used here would result in greater malformations or toxicity. Also, the cumulative effect of treatment since before the pregnancy period would refer to a treatment model closer to that used therapeutically, unraveling possible implications on parameters related to implantation and pregnancy success. Finally, it would be important to measure whether etanercept reaches fetal circulation in this experimental model.

Therefore, taken together, these findings demonstrate that etanercept favored morphological alteration in the placenta, along with growth restriction and augmented rate of fetal malformation in Wistar rats. However, it remains a challenge to treat chronic conditions in women in reproductive age, raising the concept of safety both to mother and fetus. Studies in this area should be encouraged to further attest the use of these biological compounds during pregnancy.

## Data Availability Statement

The raw data supporting the conclusions of this article will be made available by the authors, without undue reservation.

## Ethics Statement

The animal study was reviewed and approved by the Brazilian College of Animal Experiments, the Animal Ethics Committee (CEUA #23108.038471/2019-14) of the Federal University of Mato Grosso.

## Author Contributions

GA, RP, and TS conducted the data collection. GV and GA conducted the fetal analysis. RP and FG conducted the placental morphometry. RL supervised the statistical evaluation. FG, VL, and GA conducted the hypothesis and experimental design. All authors listed have made a substantial, direct, and intellectual contribution to the text, and data discussion.

## Conflict of Interest

The authors declare that the research was conducted in the absence of any commercial or financial relationships that could be construed as a potential conflict of interest.

## Publisher’s Note

All claims expressed in this article are solely those of the authors and do not necessarily represent those of their affiliated organizations, or those of the publisher, the editors and the reviewers. Any product that may be evaluated in this article, or claim that may be made by its manufacturer, is not guaranteed or endorsed by the publisher.
